# Intermittent everolimus administration for malignant insulinoma

**DOI:** 10.1530/EDM-14-0047

**Published:** 2014-09-01

**Authors:** Chiara Baratelli, Maria Pia Brizzi, Marco Tampellini, Giorgio Vittorio Scagliotti, Adriano Priola, Massimo Terzolo, Anna Pia, Alfredo Berruti

**Affiliations:** 1 Dipartimento di Oncologia, Oncologia Medica, Università di Torino, Azienda Ospedaliero Universitaria San Luigi Gonzaga, Regione Gonzole 1010043, Orbassano, Italy; 2 SCDU Radiologia, Università di Torino, Azienda Ospedaliero Universitaria San Luigi Gonzaga, Regione Gonzole 1010043, Orbassano, Italy; 3 Dipartimento di Scienze Cliniche e Biologiche, Medicina Interna, Università di Torino, Azienda Ospedaliero Universitaria San Luigi Gonzaga, Regione Gonzole 1010043, Orbassano, Italy; 4 Dipartimento di Specialità Medico-Chirurgiche, Scienze Radiologiche e Sanità Pubblica Università di Brescia, Oncologia Medica, Azienda Ospedaliera Spedali Civili, Brescia, Italy

## Abstract

Insulinoma is a rare form of insulin-secreting pancreatic islet cell neuroendocrine (NE) tumor. The medical treatment of the malignant NE disease of the pancreas deeply changed in the last years, thanks to the introduction of new target molecules, as everolimus. Even if the exact mechanism is not actually known, one of the side effects of everolimus, hyperglycemia, has been demonstrated to be useful to contrast the typical hypoglycemia of the insulinoma. We report the case of a patient with a metastatic malignant insulinoma treated with intermittent everolimus, obtaining an important improvement in the quality of life; this suggests the necessity of preclinical studies to analyze the cellular pathways involved in insulin-independent gluconeogenesis.

**Learning points:**

- Effect of somatostatin analogs is long-lasting in the control of functioning NE tumors.
- Persistent everolimus control of hypoglycemia despite serum insulin levels and disease progression.
- Open issue: are disease progression and the increase in serum markers the only valid criteria to reject a treatment?

## Background

Insulinoma is an uncommon insulin-secreting pancreatic islet cell neuroendocrine (NE) tumor. Its estimated incidence is approximately three to four cases per million individuals per year. It can occur sporadically (∼95%) or constitute a part of multiple endocrine neoplasia type 1 (MEN1) or Von Hippel–Lindau syndrome (4–6%) (1).

Most insulinomas are benign (90% of cases); the malignant forms should be suspected when the tumor size exceeds 4 cm and when the tumor arises in the context of MEN1.

The treatment of the malignant form is challenging, and especially, the management of the hypoglycemia is difficult.

There is limited evidence about the role of traditional cytotoxic agents in the management of the syndrome, whereas somatostatin analogs may control hypoglycemia symptoms, and inhibit tumor progression in certain disease settings (2).

Recently, a better understanding of molecular pathways has provided clues for new therapeutic strategies and a variety of targeted agents have been explored in pancreatic NE tumors.

Everolimus, an inhibitor of the mechanistic target of rapamycin (MTOR), was approved by the FDA in 2011 for the treatment of progressive pancreatic NE tumors (3). This biological agent has been mostly employed after the failure of somatostatin analogs and/or systemic chemotherapies. However, everolimus may be considered earlier in the course of treatment in presence of intolerability or contraindication for other therapies.

In a recent study, everolimus was compared with placebo in 410 patients with progressive well- to moderately- differentiated pancreatic NE tumors. Everolimus significantly prolonged disease-free interval by 6.4 months compared with placebo, and this effect was long lasting with 35% stabilizations at 18 months (4).

As the activation of the PI3K/AKT/MTOR pathway is essential for inhibiting gluconeogenesis by insulin (5), hyperglycemia is a frequent side effect of everolimus therapy, and this finding makes the drug appealing to manage insulinoma (6) (7). The exact mechanisms by which everolimus can control hypoglycemia in patients with insulinoma are not fully elucidated but are reportedly independent of tumor regression (6).

In a recent small series, hypoglycemia improvement after everolimus was associated with a reduction in serum insulin levels (7).

There are several open questions about the management of insulinoma, including the correlation among insulin serum levels, control of the hypoglycemia, and tumor response. In this paper, we report the oncological history of a patient with inoperable, malignant insulinoma treated with everolimus.

The observation of the maintenance of drug activity in controlling hypoglycemia irrespective of increasing insulin levels and the resumption of the glycemic control, which became refractory to drug administration after temporary drug withdrawal, provide clues for reflection on the mechanisms of everolimus activity and the best schedule for drug administration in the management of insulinoma.

## Case report

A 60-year-old man was diagnosed in July 2006 with liver lesions; the cytology obtained from a fine needle aspiration (FNA) indicated the presence of a metastatic lesion from NE pancreatic tumor, G2; chromogranin A serum levels were normal (73 ng/ml). A diagnosis of sporadic insulinoma was made due to frequent episodes of symptomatic hypoglycemia.

Owing to a positive single-photon emission computerized tomography (SPECT) scintigraphy for the presence of the receptors of somatostatin on pancreatic and liver lesions, the patient started a treatment with octreotide LAR 20 mg every 28 days, then increased after two years to 30 mg every 28 days obtaining a disease stabilization and glycemic control lasting for 3 years.

In May 2009, a liver progression was detected and bevacizumab in association with metronomic capecitabine was introduced (phase 2 trial, no. NCT01203306). The CT scan performed after 6 months showed a stable disease. One month later, the patient suddenly developed a symptomatic hypoglycemic episode during the night.

Blood measurements revealed low glucose levels (30 mg/dl) and elevated insulin levels (>150 mUI/ml). Treatment with i.v. glucose, prednisone, diazoxide, and high-dose octreotide LAR (60 mg) was immediately instituted. Recombinant glucagon was administered on demand. Despite this therapy, hypoglycemia was not controlled and the patient was compelled to consume frequent, small, carbohydrate-rich meals, and nocturnal feeding. The patient weight increased by ∼8 kg in two months, and in March 2010 he was subjected to radio-labeled Lu-177 octreotate treatment.

After three cycles of radionuclide treatment, a CT scan showed a stable disease but hypoglycemic symptoms were still uncontrolled and the patient presented a cushingoid habit caused by prednisone.

**Figure 1:**
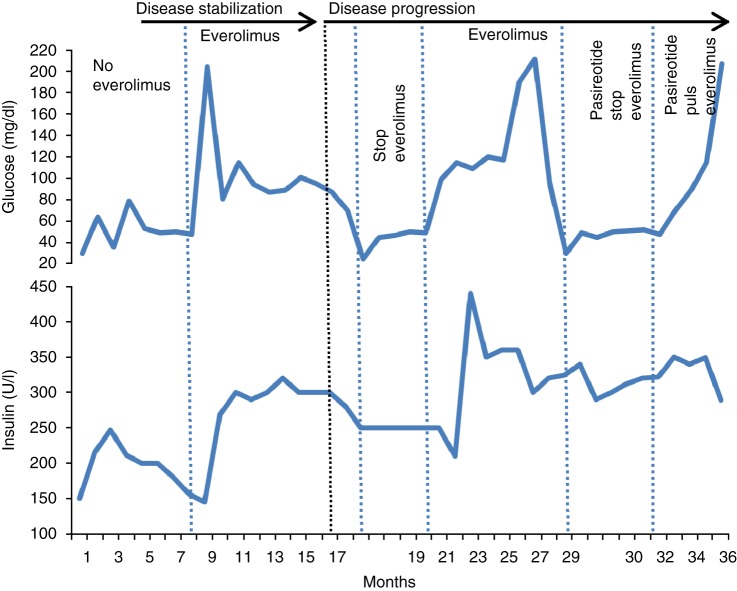
Serial evaluation of serum glucose (normal range, 70–110 mg/dl) and serum insulin (normal range, 6–29 μUI/ml) in a patient with malignant insulinoma before and during intermittent everolimus administration. The patient was hospitalized during everolimus off periods to receive i.v. glucose, prednisone, diazoxide and recombinant glucagon infusion.

In November 2010, everolimus (10 mg daily) was introduced in combination with radionuclide treatment. Glycemic control improved rapidly leading to withdrawal of diazoxide and prednisone. As reported in Fig. 1, glucose levels were normalized during everolimus therapy even if insulin levels remained persistently elevated.

Disease stabilization lasted till February 2012, when a CT scan showed progression. At that time, insulin levels increased consistently although the patient was not symptomatic. Everolimus therapy was not interrupted and hypoglycemia was controlled irrespective of disease progression and further increase in insulin levels (Fig. 1). In April 2012, the patient's clinical conditions worsened dramatically, resulting in remarkable lowering of blood glucose and severe hypoglycemia, especially during the night.

The patient was hospitalized; everolimus was discontinued and treatment with repeated infusion of glucose solution and glucagon (6 mg at 6.6 ml/h from 1100 to 0700 h by i.v. pump) was commenced.

Owing to the patient's general worsening conditions, with a low-grade renal failure, we decided not to use streptozotocin but to start chemotherapy with weekly carboplatin, area under the curve 2; it was introduced at disease progression but was totally inefficacious and was interrupted after 3 months. Everolimus was reintroduced after a 2 month withdrawal and a resumption of serum glycemia was obtained in two weeks leading to the patient's discharge from the hospital. The glycemic control lasted for eight months, during which serum insulin levels showed an increasing trend and the disease demonstrated a slow progression.

New hypoglycemic episodes occurred in November 2012, necessitating hospitalization. Everolimus was again stopped and pasireotide, a drug targeting four out of five somatostatin receptors, was introduced but it was unable to control the syndrome. Everolimus was reintroduced after a 2 months withdrawal and a glycemic control was immediately obtained, lasting for ∼2 months. At the beginning of January 2013, the patient was hospitalized again owing to acute kidney failure and he finally died after a few days, 36 months from the onset of the syndrome.

## Discussion

The management of insulinoma is challenging. The use of somatostatin analogs may not always be efficacious. The inadequate control of insulin secretion by somatostatin analogs is probably a consequence of the low expression of SSTR2 and SSTR5 in insulinomas, which also explains the reason for the low percentage of SPECT scintigraphy-positive tumors. However, malignant insulinomas, as opposed to benign ones, often express SSTR2 (8), which can be targeted therapeutically by long-acting somatostatin analogs (9). Accordingly, the patient described herein, bearing malignant insulinoma, obtained a long-lasting (three years) control of the syndrome with a somatostatin analog.

In patients with refractory syndrome to somatostatin analogs, the control of hypoglycemia was very difficult until two years ago when everolimus was introduced. This drug is actually considered a valid therapeutic option in the treatment of pancreatic NETs and is the reference drug in the management of malignant insulinoma. Several mechanisms are considered to explain the efficacy of everolimus in controlling insulinoma-induced hypoglycemia. They include a decrease in insulin production and/or an increase in peripheral insulin resistance. In our case, everolimus controlled the glucose level in spite of the high insulin level and disease progression. This underlines that the drug may be effective in the management of insulinoma irrespective of its antineoplastic activity and its effect on insulin production.

**Figure 2:**
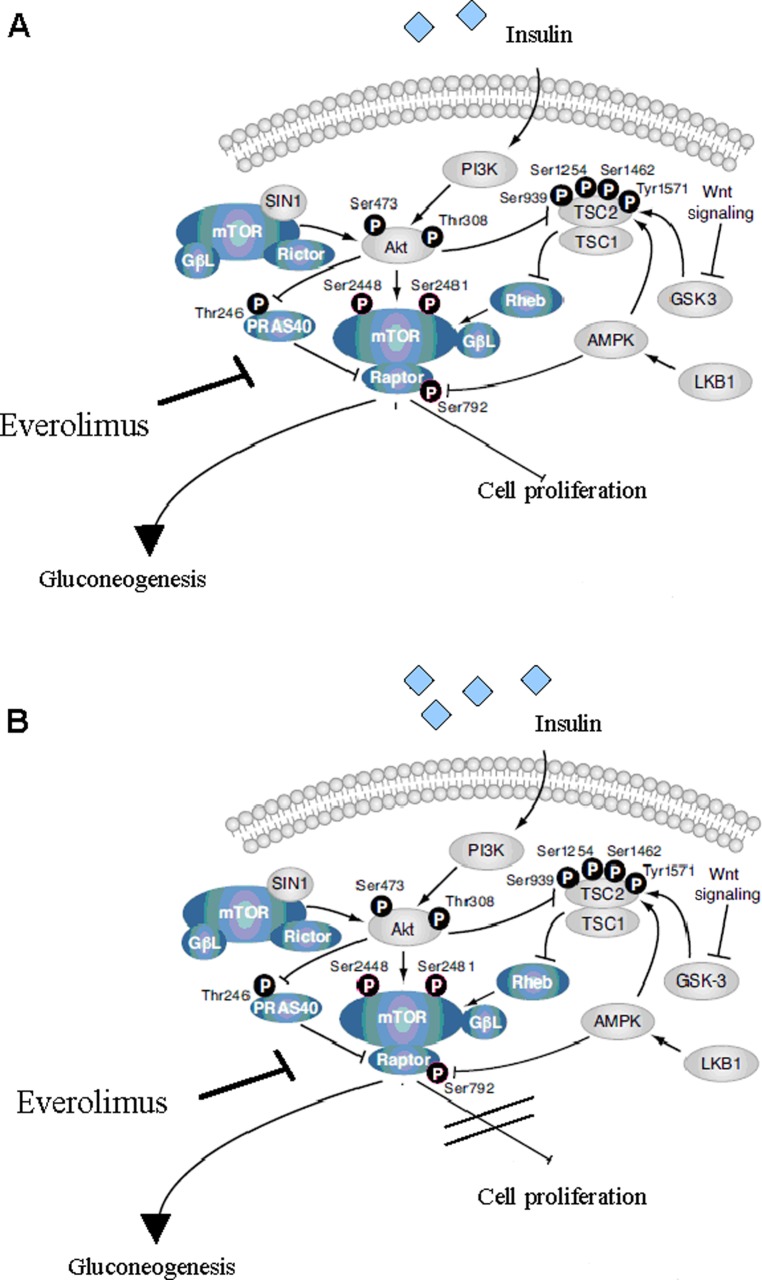
(A) mTOR pathway blockade with everolimus. (B) mTOR-induced gluconeogenesis, as a phenomenon independent of the control of tumor growth.

As the PI3K/AKT/MTOR pathway is essential for hepatic gluconeogenesis (5), everolimus may counteract hypoglycemia independent of its anti-tumor effect by enhancing the expression of liver gluconeogenic enzymes and nuclear recruitment of gluconeogenic transcriptional regulators (Fig. 2).

In a recent preclinical study, *FAM3A*, a new cytokine-like gene, activates the AKT/PI3K pathway despite insulin levels, activating the hepatic gluconeogenesis in an insulin-independent manner (10).

Everolimus reintroduction, after a temporary withdrawal, was able to resume the glycemic control that became refractory to the drug administration. Loss of the control of hypoglycemia after an initial response to everolimus, therefore, seems to be due to desensitization or downregulation of mediators involved in the MTOR pathway instead of a true resistance and can be reversed after a period of treatment interruption.

Everolimus administration in our patient resulted in disease stabilization lasting for six months, while the control of glycemia persisted for 36 months with intermittent administration.

In conclusion, despite the limitations of a single case report, the clinical history described suggests that disease progression and increasing levels of serum insulin may not be considered the only criterion for discontinuing everolimus therapy whenever hypoglycemia is controlled. Preclinical and clinical studies are required to better understand the pathways involved in glycemic control after everolimus administration as well as the potential efficacy of everolimus reintroduction after temporary withdrawal.

## Patient consent

The patient is deceased.

## Author contribution statement

All authors have contributed to the care of the patient, the management of the therapy, and the production of this work.
